# One‐year cost‐effectiveness of callosotomy vs vagus nerve stimulation for drug‐resistant seizures in Lennox‐Gastaut Syndrome: A decision analytic model

**DOI:** 10.1002/epi4.12570

**Published:** 2022-01-17

**Authors:** Taylor J. Abel, Madison Remick, William C. Welch, Kenneth J. Smith

**Affiliations:** ^1^ Department of Neurological Surgery University of Pittsburgh Pittsburgh Pennsylvania USA; ^2^ Department of Bioengineering University of Pittsburgh Pittsburgh Pennsylvania USA; ^3^ Division of Pediatric Neurology Department of Pediatrics University of Pittsburgh Pittsburgh Pennsylvania USA; ^4^ Department of Medicine University of Pittsburgh Pittsburgh Pennsylvania USA

**Keywords:** corpus callosotomy, drug‐resistant epilepsy, epilepsy surgery, Lennox‐Gastaut Syndrome, vagus nerve stimulator

## Abstract

**Objective:**

Palliative epilepsy surgery via corpus callosotomy (CC) or vagus nerve stimulation (VNS) is commonly employed for drug‐resistant seizures in Lennox‐Gastaut Syndrome (LGS). VNS is less effective at reducing seizures but has fewer adverse events, CC is more effective for seizure control, particularly atonic seizures, but can be associated with serious adverse events, and yet their relative cost‐effectiveness remains unknown.

**Methods:**

To determine which option is most cost‐effective, a decision analytic model was developed to evaluate the risks and benefits of CC and VNS at 1 year based on costs in the United States. Our primary outcome measure was positive seizure outcomes, defined as >50% seizure reduction without procedural complications.

**Results:**

CC had a 15% greater likelihood of a positive seizure outcome, but per patient costs were $68 147 more than VNS, or $451 952 per positive seizure outcome gained. One‐way sensitivity analyses demonstrate that probabilities of seizure freedom or reduction by VNS or CC and CC cost were most influential on results. When considering atonic seizures, CC had a 27% greater positive outcome likelihood than VNS, the same incremental cost, and cost $250 556 per positive seizure outcome gained.

**Significance:**

This exploratory model suggests that VNS is more cost‐effective relative to CC at 1 year.

**Key Points**

- Decision analysis shows that corpus callosotomy is associated with higher rates of seizure control, vagus nerve stimulation is a more cost‐effective option.
- The incremental cost‐effectiveness of CC relative to VNS was $451 952 per positive seizure outcome gained.
- VNS is more cost‐effective relative to CC and may be considered a first‐line palliative surgery option.

## 1 INTRODUCTION

Lennox‐Gastaut syndrome (LGS) is a severe form of epilepsy characterized by childhood onset, multiple drug‐resistant seizure types, and long‐term poor prognosis.^1^ Given the difficulty controlling seizures in LGS, vagus nerve stimulation (VNS) and corpus callosotomy (CC) are both often considered palliative surgical options.^2^ However, there is no current consensus about which procedure is superior or what procedure should be performed first.^3^, ^4^ Furthermore, while cost‐benefit and cost‐utility analyses suggest decreased healthcare utilization costs in LGS‐like epilepsy after palliative epilepsy surgery,^5^, ^6^ less attention has been paid to the relative cost‐effectiveness of CC vs VNS. It is generally accepted that CC is more effective for seizure reduction than VNS,^7^ particularly for atonic seizures which are characteristic of LGS.^7^, ^8^ However, VNS is considered lower risk, but potentially less cost‐effective due to device costs and need for periodic device revisions.^3^, ^4^, ^9^ Understanding the relative risks, benefits, and cost‐effectiveness of CC and VNS may shed light on this clinical conundrum and inform prudent surgical decision‐making for patients with LGS. Thus, we developed a simple decision analytic model to investigate the relative cost‐effectiveness of CC and VNS over a 1‐year time horizon. As a base case, we considered an LGS patient deemed a candidate for palliative epilepsy surgery via CC or VNS.

## 2 METHODS

### 2.1 Decision model

**FIGURE 1:**
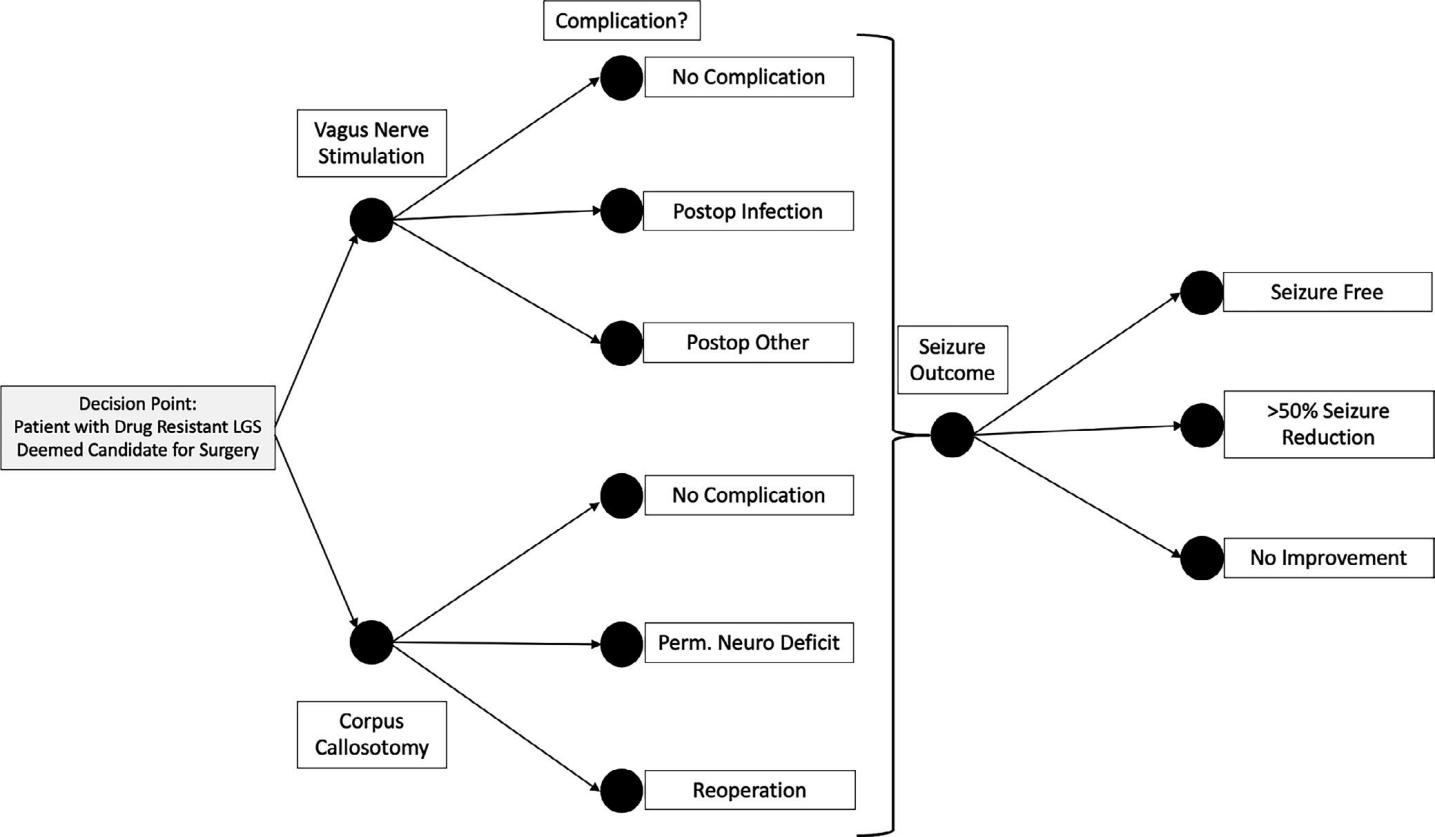
Decision Analytic Model. Probabilities are assigned to each chance node based on the parameters described in Table 1

We followed the Consolidated Health Economic Evaluation Reporting Standards (CHEERS) statement as a guideline to perform this study.^10^ We developed a decision analytic model to compare CC to VNS as a treatment for drug‐resistant seizures in LGS considering a healthcare perspective (Figure 1). Based on CHEERS guidelines, the base case of a patient in the United States with drug‐resistant LGS‐type epilepsy was considered for this model, based on clinical parameters obtained from available literature (see Clinical Variables). For this model, a 1‐year time horizon was utilized to account for the risks and benefits of each procedure up to 1 year but would obviate the complexities introduced by a longer term model. The modeled cohort consisted of hypothetical patients with LGS and drug‐resistant seizures^11^ (ie, failed two or more anti‐seizure medications^11^) with multiple seizure types being considered for palliative epilepsy surgery. This model considers a decision between either VNS or CC (Figure 1). For each procedure, important complications relevant to each procedure were considered, including postoperative infection and miscellaneous for VNS and permanent or reoperation (eg, for hemorrhage) for CC (Figure 1). In this analysis, we did not distinguish between different callosotomy techniques, such as anterior two‐thirds callosotomy vs complete callosotomy.^12^ Additionally, costs were considered only for traditional open callosotomy approaches rather than radiosurgical callosotomy or stereotactic laser ablation callosotomy.^13^, ^14^

For the purpose of this analysis, an outcome of at least >50% seizure reduction and no complication were considered positive. In contrast, no improvement in seizures or occurrence of any complication was considered adverse. Common complications that may be considered adverse outcomes were included in the model. For CC, permanent neurologic deficit and reoperation were considered adverse outcomes. For VNS, infection and other VNS complications (ie, need for lead revision) were considered adverse outcomes. Seizure freedom, >50% seizure reduction, and no improvement in seizures were considered discrete outcomes. Seizure freedom or >50% seizure reduction was considered positive outcomes, while no improvement in seizures was considered an adverse outcome.

### 2.2 Clinical variables

Table 1 summarizes the clinical variables and data sources used as inputs for the decision analysis. Clinical outcomes and complication probabilities were obtained from recent systematic reviews that synthesize and compare CC and VNS literature.^8^, ^15^ Given that LGS patients have multiple seizure types, seizure reduction probabilities were considered as an average of seizure reduction from all seizure types.^8^ Seizure freedom and >50% seizure reduction were considered as discrete probabilities; thus, rates of seizure freedom were subtracted from rates of >50% seizure reduction to calculate the probabilities of seizure freedom and >50% seizure reduction.

**TABLE 1:** Clinical Parameters and Data Sources Used in Decision Analysis

Variable	Value	Range	Reference
Cost estimates			
Callosotomy	$92 800	$72 900‐178 800	Oldham et al, Ped Neurol 2015.
VNS	$30 091	$20 000‐40 000	De Kinderen et al, Epilepsy Res 2015. Oldham et al, Ped Neurol 2015.
VNS infection	N/A	$0‐100 000	Wide estimate for sensitivity analysis
Other VNS complications	N/A	$0‐100 000	Wide estimate for sensitivity analysis
Permanent neurologic deficit	N/A	$0‐500 000	Wide estimate for sensitivity analysis
Reoperation	N/A	$0‐500 000	Wide estimate for sensitivity analysis
Clinical parameters^a^			
Seizure reduction after CC	47.0%	38.0%‐56.0%	Lancman et al, Seizure 2013.
Seizure free after CC	16.0%	10.0%‐23.0%	Lancman et al, Seizure 2013.
Seizure reduction after VNS	44.1%	32.3%‐56.0%	Lancman et al, Seizure 2013.
Seizure free after VNS	5.2%	2.3%‐9.2%	Lancman et al, Seizure 2013.
Atonic seizure reduction after CC	32.0%	19.0%‐42.0%	Lancman et al, Seizure 2013.
Atonic seizure free after CC	48.0%	31.0%‐65.0%	Lancman et al, Seizure 2013.
Atonic seizure reduction after VNS	31.3%	9.3%‐49.6%	Lancman et al, Seizure 2013.
Atonic seizure free after VNS	22.8%	6.6%‐44.9%	Lancman et al, Seizure 2013.
Probability of permanent neurologic deficit	0.6%	0.6%‐5.0%	Ye et al, Childs Nerv Sys 2021. Lancman et al, Seizure 2013.
Probability of VNS infection	2.4%	1.5%‐2.4%	Ye et al, Childs Nerv Sys 2021.
Probability of other VNS complications	1.4%	1.4%‐2.8%	Ye et al, Childs Nerv Sys 2021.
Probability of reoperation after CC	6.6%	6.6%‐10.0%	Ye et al, Childs Nerv Sys 2021.

^a^ Seizure freedom and >50% seizure reduction were considered as discrete probabilities; thus, the rates of seizure freedom were subtracted from the rate of >50% seizure reduction to calculate the probabilities of seizure freedom and >50% seizure reduction.

Costs were obtained from available cost utility literature for each surgery and reflect cost in US dollars.^16^, ^17^ Costs for VNS infection and CC reoperation were estimated as the same as the index procedure. Given that VNS complication and CC complication costs are not documented extensively in the literature, we varied these parameters widely in subsequent sensitivity analyses. We assumed that baseline healthcare utilization costs not related to the surgical procedures would be similar for CC and VNS as a function of whether seizures improved, so these costs were not included in the model.

### 2.3 Cost‐effectiveness analysis

Cost‐effectiveness analysis was performed with regard to cost per positive outcome (ie, seizure improvement) in the main analysis. Analyses were performed from a healthcare perspective, which considers direct medical costs. Strategies were compared using the incremental cost‐effectiveness ratio (ICER), the difference in costs between strategies divided by the difference in positive outcome likelihood. Given a 1‐year time horizon, discounting was not performed and a discount rate was not considered.

### 2.4 Sensitivity analyses

We performed one‐way sensitivity analyses, individually varying parameters to account for uncertainty in clinical variables and to detect how variation influences model results. Once one‐way sensitivity analyses were performed, a two‐way sensitivity analysis was also performed for the two most influential clinical parameters to determine influence on model outcome. While the base case analysis examined LGS patients with multiple seizure types, another analysis specifically examined CC and VNS outcomes for atonic seizures. This additional scenario analysis was performed since many centers perform CC specifically for atonic seizures and outcome differences for this seizure subtype could have important implications for the interpretation of model results.

## 3 RESULTS

### 3.1 Base case cost‐effectiveness analysis

Base case analysis demonstrated that CC had a 15% greater likelihood of a positive seizure outcome (ie, seizure freedom or >50% seizure reduction), but an incremental per patient cost that was $68 147 more than VNS (Table 1). The incremental cost‐effectiveness of CC relative to VNS was $451 952 per positive seizure outcome gained.

### 3.2 One‐way and two‐way sensitivity analyses

**FIGURE 2:**
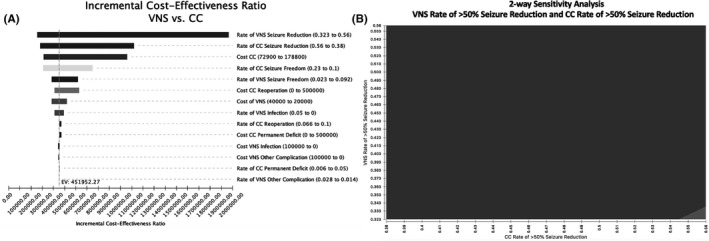
(A) Results of One‐way Sensitivity Analysis. Tornado diagram depicting results of one‐way sensitivity analyses. (B) Results of Two‐Way Sensitivity Analysis. Two‐way sensitivity analysis varying rate of seizure reduction by VNS and rate of seizure reduction by CC considering a willingness‐to‐pay of $200 000 per positive seizure outcome gained. The red area denotes parameter values where VNS is favored; blue denotes where CC is favored

One‐way sensitivity analyses (Figure 2A) showed that variation in respective rates of seizure reduction, or freedom by each treatment modality, had the greatest influence on model results. The likelihood of >50% seizure reduction with VNS had the greatest influence on results, with the likelihood of >50% seizure reduction by CC, CC cost, and CC‐ and VNS‐related seizure freedom also being influential (Figure 2A). Interestingly, despite strong consideration for differences in complication rates between CC and VNS, results were relatively insensitive to variation of complication rates.

A two‐way sensitivity analysis was performed for the two most influential model variables: rates of seizure reduction by VNS and by CC (Figure 2B). When considering a willingness to pay of $200 000, VNS remained the favored option in nearly every possible scenario. CC was considered favorable when the probability of >50% seizure reduction by CC is greater than 55% and the rate of >50% seizure reduction by VNS is less than 34%. In summary, both one‐way and two‐way sensitivity analyses reveal few scenarios, in which CC would be more cost‐effective than VNS.

### 3.3 Scenario analysis: Atonic seizures

In a separate scenario analysis, CC and VNS outcomes were compared specifically for atonic seizures (Table 2). This analysis was performed given literature suggesting that atonic seizures are the primary indication for CC.^18^, ^19^ When the model was performed specifically for atonic seizures, CC had a 27% greater likelihood of a good outcome than VNS and the same incremental cost per patient (ie, $68 147). Compared to when the model considered all seizure types, CC had a more favorable incremental cost compared to VNS of $250 556 per positive seizure outcome gained. Thus, VNS remained more economically reasonable relative to CC for atonic seizures (Table 2).

**TABLE 2:** Base case cost‐effectiveness analysis

Strategy	Cost	Incremental cost	Effectiveness^a^	Incremental Effectiveness	ICER
Base case cost‐effectiveness analysis					
VNS	$30 844	—	0.48	—	—
CC	$98 991	$68 147	0.63	0.15	$451 952
Atonic seizure cost‐effectiveness analysis					
VNS	$30 844	—	0.53	—	—
CC	$98 991	$68 147	0.80	0.27	$250 556

Abbreviation: ICER, incremental cost‐effectiveness ratio.

^a^ Effectiveness is the probability of a positive outcome.

## 4 DISCUSSION

Our results show that while CC is associated with higher rates of seizure control, VNS is a more cost‐effective approach at 1 year. This finding was robust despite wide variation in model input parameters. Existing literature depicts both CC and VNS as promising therapies for LGS,^1^ but there is no consensus on whether to offer CC or VNS first.^3^ We show that given common CC and VNS risk‐benefit considerations in the average patient, VNS is likely the most efficient first option in LGS patients. This interpretation was true even when considering atonic seizures specifically. Complication rates are often cited as rationale for considering VNS first,^3^ but our sensitivity analyses show that complication rates have the least influence on model results.

We considered “positive” vs “adverse” outcomes in a binary fashion, where a “positive” outcome was considered >50% seizure reduction without complication and any other outcome was “adverse.” There is undoubtedly a range of potential outcomes that could be modeled if considering quality‐adjusted life years (QALYs). This would be important when considering more subtle cognitive outcomes of CC in combination with long‐term seizure control, particularly in higher functioning LGS patients. Well‐designed prospective studies will be necessary to generate the data required to rigorously perform these more granular analyses.

Our model considered 1‐year outcomes of CC or VNS, without considering changes in baseline healthcare utilization or long‐term cost of routine VNS revisions. We assumed that changes in healthcare utilization would be similar in the setting of positive seizure outcome of either surgery. Though partially accounted for by sensitivity analysis, the model does not adequately account for the long‐term costs of rare and serious procedural complications. Further, this model does not account for costs accrued from VNS programming visits. Pulse generator revisions, which are not accounted for in this model, are required approximately every 5 years after VNS implantation. Assuming an operation cost of approximately $40 000 and pulse generator revisions every 5 years, the ICERs shown in our analysis would be expected to show that VNS continues to be cost‐effective relative to CC for approximately 20 years for atonic seizures and longer for other seizure types for which callosotomy is less effective. Additionally, this model does not account for potential recurrence or worsening of seizures after CC or VNS, which would be important to consider in models with a longer term time horizon. That said, if the effectiveness of CC decreased over time, while the effectiveness of VNS increases over time, then the model would continue to favor VNS.

This analysis considers costs per positive seizure outcome gained; however, there is no benchmark value for what a positive seizure outcome might be worth, a potential limitation. Despite this, results favoring VNS were robust in analyses using thresholds of ≤$200 000 per positive outcome. Future highly detailed analyses, using QALYs with commonly cited benchmarks as the effectiveness term in a cost‐effectiveness analysis should provide greatly clarity. Based on our results, VNS is a more cost‐effective strategy for LGS patients when considering outcomes at 1 year; however, treatment decisions should also consider alterations in quality of life, baseline healthcare utilization costs, and long‐term device costs. An important consideration not accounted for by this model is the influence of seizure control on cognitive development. Earlier seizure control resulting from palliative epilepsy surgery may be associated with improved cognitive outcomes, which would favor the more effective strategy (CC).

## 5 CONCLUSIONS

According to our exploratory model, VNS is a more cost‐effective option relative to CC for LGS patients with drug‐resistant seizures when considering 1‐year outcomes and costs in USD. Future directions include more complex cost‐effectiveness models that consider longer time horizons and comparative effectiveness studies that provide more detailed cost, outcome, and quality of life data. Future cost‐effectiveness studies should consider alterations in quality of life, baseline healthcare utilization costs, and long‐term device costs.

## CONFLICT OF INTEREST

TJA is a consultant for and receives research funding from the Monteris Medical Corporation. MR, WCW, and KJS have no conflicts of interest to disclose. We confirm that we have read the Journal's position on issues involved in ethical publication and affirm that this report is consistent with those guidelines.

## References

[1] Arzimanoglou A , French J , Blume WT , Cross JH , Ernst J‐P , Feucht M , et al. Lennox‐Gastaut syndrome: a consensus approach on diagnosis, assessment, management, and trial methodology. Lancet Neurol. 2009;8:82–93. 19081517 10.1016/S1474-4422(08)70292-8

[2] Nei M , O'Connor M , Liporace J , Sperling MR . Refractory generalized seizures: response to corpus callosotomy and vagal nerve stimulation. Epilepsia. 2006;47:115–22. 10.1111/j.1528-1167.2006.00377.x 16417539

[3] Rosenfeld WE , Roberts DW . Tonic and atonic seizures: what's next–VNS or callosotomy? Epilepsia. 2009;50:25–30. 10.1111/j.1528-1167.2009.02232.x 19702730

[4] Wong T‐T , Kwan S‐Y , Chang K‐P , Hsiu‐Mei WU , Yang T‐F , Chen Y‐S , et al. Corpus callosotomy in children. Child's Nerv Syst. 2006;22:999–1011. 16830167 10.1007/s00381-006-0133-4

[5] Forbes R . Cost‐utility of vagus nerve stimulation (VNS) therapy for medically refractory epilepsy–an update. Seizure. 2008;17:387–8. 18584780 10.1016/j.seizure.2007.10.005

[6] Helmers SL , Duh MS , Guérin A , Sarda SP , Samuelson TM , Bunker MT , et al. Clinical outcomes, quality of life, and costs associated with implantation of vagus nerve stimulation therapy in pediatric patients with drug‐resistant epilepsy. Eur J Paediatr Neurol. 2012;16:449–58. 22261080 10.1016/j.ejpn.2012.01.001

[7] Cukiert A , Cukiert CM , Burattini JA , Lima AM , Forster CR , Baise C , et al. Long‐term outcome after callosotomy or vagus nerve stimulation in consecutive prospective cohorts of children with Lennox‐Gastaut or Lennox‐like syndrome and non‐specific MRI findings. Seizure. 2013;22:396–400. 23490456 10.1016/j.seizure.2013.02.009

[8] Lancman G , Virk M , Shao H , Mazumdar M , Greenfield JP , Weinstein S , et al. Vagus nerve stimulation vs. corpus callosotomy in the treatment of Lennox‐Gastaut syndrome: a meta‐analysis. Seizure. 2013;22:3–8. 23068970 10.1016/j.seizure.2012.09.014 PMC3655762

[9] Fandiño‐Franky J , Torres M , Nariño D , Fandiño J . Corpus callosotomy in Colombia and some reflections on care and research among the poor in developing countries. Epilepsia. 2000;41:22–7. 10.1111/j.1528-1157.2000.tb01541.x 10963473

[10] Husereau D , Drummond M , Petrou S , Carswell C , Moher D , Greenberg D , et al. Consolidated health economic evaluation reporting standards (CHEERS) statement. Eur J Heal Econ. 2013;16:e1–5. 10.1016/j.jval.2013.02.010 23538200

[11] Kwan P , Arzimanoglou A , Berg AT , Brodie MJ , Allen Hauser W , Mathern G , et al. Definition of drug resistant epilepsy: consensus proposal by the ad hoc task force of the ILAE commission on therapeutic strategies. Epilepsia. 2010;51:1069–77. 19889013 10.1111/j.1528-1167.2009.02397.x

[12] Kasasbeh AS , Smyth MD , Steger‐May K , Jalilian L , Bertrand M , Limbrick DD . Outcomes after anterior or complete corpus callosotomy in children. Neurosurgery. 2014;74:17–28. 24089047 10.1227/NEU.0000000000000197

[13] Feichtinger M , Schrottner O , Eder H , Holthausen H , Pieper T , Unger F , et al. Efficacy and safety of radiosurgical callosotomy: a retrospective analysis. Epilepsia. 2006;47:1184–91. 16886982 10.1111/j.1528-1167.2006.00592.x

[14] Huang Y , Yecies D , Bruckert L , Parker JJ , Ho AL , Kim LH , et al. Stereotactic laser ablation for completion corpus callosotomy. J Neurosurg Pediatr. 2019;2:1–9. 10.3171/2019.5.PEDS19117 31374542

[15] Ye VC , Mansouri A , Warsi NM , Ibrahim GM . Atonic seizures in children: a meta‐analysis comparing corpus callosotomy to vagus nerve stimulation. Child's Nerv Syst. 2021;37:259–67. 32529546 10.1007/s00381-020-04698-0

[16] Oldham MS , Horn PS , Tsevat J , Standridge S . Costs and clinical outcomes of epilepsy surgery in children with drug‐resistant epilepsy. Pediatr Neurol. 2015;53:216–20. 26138748 10.1016/j.pediatrneurol.2015.05.009

[17] de Kinderen RJA , Postulart D , Aldenkamp AP , Evers SMAA , Lambrechts DAJE , Louw AJAD , et al. Cost‐effectiveness of the ketogenic diet and vagus nerve stimulation for the treatment of children with intractable epilepsy. Epilepsy Res. 2015;110:119–31. 25616464 10.1016/j.eplepsyres.2014.12.005

[18] Thohar Arifin M , Muttaqin Z , Bakhtiar Y , Andar E , Priambada D , Kurnia H , et al. Seizure outcomes in patients with complete versus anterior corpus callosotomy: analysis of outcome. Int J Gen Med. 2020;13:105–10. 32280261 10.2147/IJGM.S247438 PMC7127778

[19] Iwasaki M , Uematsu M , Sato Y , Nakayama T , Haginoya K , Osawa S‐I , et al. Complete remission of seizures after corpus callosotomy: clinical article. J Neurosurg Pediatr. 2012;10:7–13. 22681320 10.3171/2012.3.PEDS11544

